# Exploring the Interplay of Humor and Quality of Life in Adults Confronting Chronic Diseases: A Comprehensive Systematic Review

**DOI:** 10.1192/j.eurpsy.2024.548

**Published:** 2024-08-27

**Authors:** E. Bartzou, E. Tsiloni, S. Mantzoukas, E. Dragioti, M. Gouva

**Affiliations:** ^1^Research Laboratory Psychology of Patients, Families & Health Professionals, Department of Nursing, School of Health Sciences, University of Ioannina, Ioannina; ^2^Department of Educational Sciences and Social Work, University of Patras, Patra; ^3^Research Laboratory of Integrated Health, Care and Well-being, Department of Nursing, School of Health Sciences, University of Ioannina, Ioannina, Greece

## Abstract

**Introduction:**

Chronic diseases, often referred to as non-communicable diseases (NCDs), stand as the leading global cause of mortality. Individuals grappling with chronic ailments frequently experience a decline in their overall quality of life (QoL), encompassing psychological, social, and physical dimensions of well-being.

**Objectives:**

Recognizing that humor has demonstrated the potential to engender favorable effects on QoL, this systematic review seeks to explore the correlation between humor and QoL among adults contending with chronic health conditions.

**Methods:**

A thorough examination of quantitative data was conducted in strict adherence to the PRISMA 2020 guidelines. PubMed/MEDLINE, PsycINFO, and CINAHL were comprehensively searched from their inception until June 22, 2023. Furthermore, the reference lists of the included datasets and relevant review articles were exhaustively scrutinized (Figure 1). The Newcastle-Ottawa Scale (NOS) was employed to assess the quality of eligible studies.

**Results:**

A total of eighteen studies met the inclusion criteria, encompassing a diverse spectrum of chronic disease categories, including cardiovascular diseases and various types of cancer, among others. Collectively, these studies involved a participant cohort comprising 4,325 individuals. Noteworthy findings emerged, indicating a substantial association between distinct facets of humor—such as one’s sense of humor, coping humor, humor styles, and laughter—and psychological QoL. Nevertheless, the relationship between humor and physical QoL exhibited a more intricate pattern, characterized by mixed outcomes (Figure 2).

**Image:**

**Figure:**
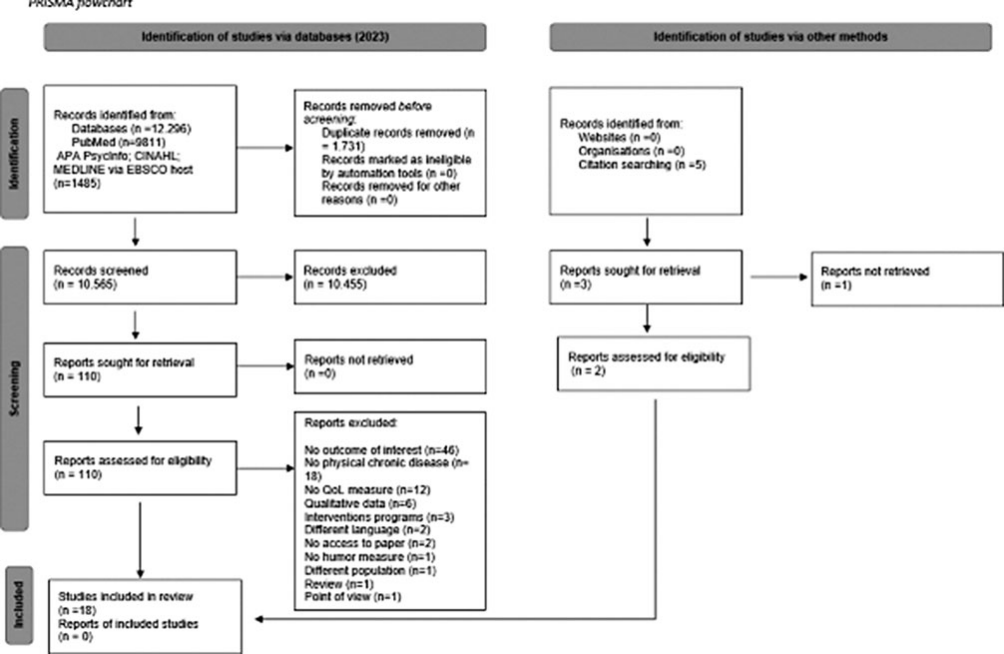

**Image 2:**

**Figure:**
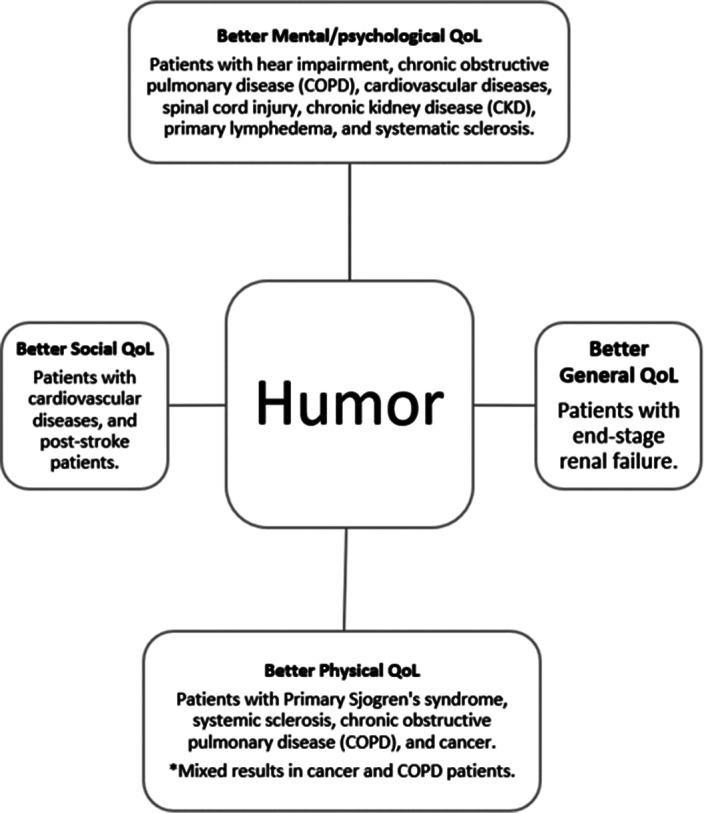

**Conclusions:**

Despite the limited and inconsistent evidence across studies, humor appears to exhibit a positive association with QoL.

**Disclosure of Interest:**

None Declared

